# The CDKN2A G500 Allele Is More Frequent in GBM Patients with No Defined Telomere Maintenance Mechanism Tumors and Is Associated with Poorer Survival

**DOI:** 10.1371/journal.pone.0026737

**Published:** 2011-10-26

**Authors:** Janice A. Royds, Shafagh Al Nadaf, Anna K. Wiles, Yu-Jen Chen, Antonio Ahn, Alisha Shaw, Sara Bowie, Frederic Lam, Bruce C. Baguley, Antony W. Braithwaite, Martin R. MacFarlane, Noelyn A. Hung, Tania L. Slatter

**Affiliations:** 1 Department of Pathology, Dunedin School of Medicine, University of Otago, Dunedin, New Zealand; 2 Department of Medicine, Dunedin School of Medicine, University of Otago, Dunedin, New Zealand; 3 Zenith Technology, Dunedin, New Zealand; 4 Auckland Cancer Society Research Centre, Faculty of Medical and Health Sciences, University of Auckland, Auckland, New Zealand; 5 Children's Research Institute, University of Sydney, Westmead, Australia; 6 Christchurch Hospital, Christchurch, New Zealand; Instituto de Investigación Sanitaria INCLIVA, Spain

## Abstract

Prognostic markers for glioblastoma multiforme (GBM) are important for patient management. Recent advances have identified prognostic markers for GBMs that use telomerase or the alternative lengthening of telomeres (ALT) mechanism for telomere maintenance. Approximately 40% of GBMs have no defined telomere maintenance mechanism (NDTMM), with a mixed survival for affected individuals. This study examined genetic variants in the cyclin-dependent kinase inhibitor 2A (*CDKN2A)* gene that encodes the p16^INK4a^ and p14^ARF^ tumor suppressors, and the isocitrate dehydrogenase 1 (IDH1) gene as potential markers of survival for 40 individuals with NDTMM GBMs (telomerase negative and ALT negative by standard assays), 50 individuals with telomerase, and 17 individuals with ALT positive tumors. The analysis of *CDKN2A* showed NDTMM GBMs had an increased minor allele frequency for the C500G (rs11515) polymorphism compared to those with telomerase and ALT positive GBMs (p = 0.002). Patients with the G500 allele had reduced survival that was independent of age, extent of surgery, and treatment. In the NDTMM group G500 allele carriers had increased loss of *CDKN2A* gene dosage compared to C500 homozygotes. An analysis of *IDH1* mutations showed the R132H mutation was associated with ALT positive tumors, and was largely absent in NDTMM and telomerase positive tumors. In the ALT positive tumors cohort, *IDH1* mutations were associated with a younger age for the affected individual. In conclusion, the G500 *CDKN2A* allele was associated with NDTMM GBMs from older individuals with poorer survival. Mutations in IDH1 were not associated with NDTMM GBMs, and instead were a marker for ALT positive tumors in younger individuals.

## Introduction

Acquisition of a telomere maintenance mechanism prevents telomere attrition and is a hallmark of cancer [1], [2]. Most tumors utilize the telomerase enzyme to maintain telomere DNA repeats, and a minority use an alternative mechanism characterized by heterogeneous telomere lengths known as alternative lengthening of telomeres (ALT) [3], [4]. In the case of tumors negative for telomerase and ALT by standard assays (non defined telomere maintenance mechanism, NDTMM), it is unclear if no telomere maintenance occurs, if telomerase activity is below the detection limit of the current assays, or if telomeres are maintained by an unrecognized mechanism.

In glioblastoma multiforme (GBM) all of the telomere maintenance scenarios outlined above occur [5]. Individuals with ALT positive GBMs have an improved prognosis compared to non-ALT GBMs. Within the ALT positive GBM group the prognosis is better for those individuals with mutations in the tumor protein p53 (TP53) gene, and mutations in the isocitrate dehydrogenase 1 gene [5], [6], [7], [8]. In telomerase positive tumors *TP53* mutations are a marker for individuals with a poorer prognosis [5], [7], [8]. To date no prognostic markers have been identified for the approximately 40% of patients whose GBMs are without a currently defined telomere maintenance mechanism.

Other molecular characteristics of GBMs include loss of the cyclin-dependent kinase inhibitor 2A (*CDKN2A)* that encodes two proteins p16^INK4a^ and p14^ARF^ [9], [10], [11]. The most frequent polymorphism in *CDKN2A*, a substitution in the 3′ UTR of a cytosine for a guanine base at cDNA nucleotide 500 (numbered from the p16^INK4a^ initiation codon, rs11515), is associated with different cancer types. The G500 allele had an increased frequency in melanoma families, and in psoriasis patients with squamous cell carcinoma [12], [13], [14]. The G500 allele predicted a shorter progression time from primary to metastatic melanoma, a reduced tumor free survival period in individuals with bladder carcinoma, and was associated with loss of *p16^INK4a^* expression in sporadic colorectal cancer [15], [16], [17]. Functional evaluation of the C500G polymorphism is in its infancy. This polymorphism is predicted to affect the micro RNA (MiR) 601 binding, and the G500 allele is associated with increased *CDKN2A* expression [18], [19]. The G500 allele is also associated with reduced cyclin dependent inhibitor 2B (*CDKN2B)* expression; a gene in close proximity to *CDKN2A* on chromosome 9p21 and encodes the tumor suppressor p15^INK4B^ [19].

Due to the potential importance for the *CDKN2A* C500G polymorphism and *IDH1* mutations in GBM, the current study investigated the C and G 500 allele frequencies in genomic DNA, and IDH1 mutations in tumor DNA from 107 individuals with telomerase, ALT, and NDTMM GBMs. The G500 allele was associated with NDTMM tumors and was further evaluated as a marker for reduced patient survival, and increased loss of *CDKN2A* gene dosage in tumors.

## Results

One hundred and seven GBM tumors were obtained at neurosurgical units within New Zealand. Seventeen tumors (16%) were ALT positive by standard techniques i.e. long and heterogeneous telomere lengths by TRF length analysis, the presence of large aggregates of the promyelocytic leukemia (PML) protein and telomere DNA called ALT-associated promyelocytic leukemia (PML) bodies (APBs) in >0.5% of tumor cells, and very low or no telomerase activity in tumor protein lysates by the TRAP assay [3], [8]. Fifty tumors (47%) were telomerase positive by TRAP analysis. Forty tumors (37%) were classified as telomerase activity negative based on the standard TRAP assay criteria, and negative for ALT by the absence of long heterogeneous telomeres by TRF analysis [5], [8]. Henceforth, these tumors are referred to as NDTMM. The demographic data for the ALT, NDTMM, and telomerase positive tumor groups are listed in Table 1.

**Table 1:** Demographic Characteristics of GBM Patients.

Characteristic	ALT+	NDTMM	TEL+	Significance between groups
Age years (25^th^–75^th^ percentile)	40 (34–51)	65 (51–71)	61 (48–68)	*p<0.001
Gender n (%)				ns
Male	10 (59%)	27 (54%)	28(56%)	
Female	7 (41%)	23 (46%)	22 (44%)	
Treatment n (%)	16	39	44	
Surgery only	3 (19%)	6 (15%)	6 (13%)	ns
Surgery +RT	7 (44%)	18 (46%)	21 (47%)	ns
Surgery +RT + CT	6 (38%)	14 (36%)	(21 (47%)	ns
Extent of surgery				ns
Biopsy	4 (24%)	9 (22%)	12 (24%)	
Debulking	13 (76%)	31 (78%)	38 (76%)	

*The significant difference for both the ALT+ versus NDTMM, and the ALT+ versus TEL+ GBM comparison. RT, radiotherapy; CT, chemotherapy.

### The G500 allele is associated with GBMs with no defined telomere maintenance mechanism

Individuals in each GBM telomere maintenance subgroup were genotyped for the C500G polymorphism in the 3′ *CDKN2A* UTR (rs11515). The C500G genotypes are given in Table 2. In the NDTMM tumor group 19 individuals were heterozygous and two were homozygous for the G500 allele (allele frequency 0.29), and 19 were homozygous for the C500 allele. In the telomerase positive tumor group 12 individuals were heterozygous for G500 (allele frequency 0.12), and 38 were homozygous for the C500 allele. In the ALT positive tumor group three individuals were heterozygous for G500 (allele frequency 0.09), and 14 were homozygous for the C500 allele. The G500 allele frequency was significantly higher in the NDTMM compared to the telomerase (p = 0.007) and the ALT (p = 0.03) tumor group (p = 0.002, NDTMM versus telomerase and ALT positive GBMs combined). The G500 allele was also genotyped in 150 control individuals selected from the general population (Table 1). In the general population the G500 allele frequency was 0.13, significantly lower compared to the NDTMM tumor group (p = 0.001), but not significantly different to the telomerase positive GBM group.

**Table 2:** The G500 allele is more frequent in GBMs without a definitive telomere maintenance mechanism.

Telomere Maintenance Mechanism	N	G500 allele frequency
Non telomerase-ALT	40	0.29 (19 C/G, 2 G/G, 19 C/C)**
Telomerase	50	0.12 (12 C/G, 38C/C)
ALT	17	0.09 (3 C/G, 14 C/C)
Non tumor cohort	150	0.13 (32 C/G, 3 G/G, 115 C/C)

n, number of individuals; non-tumor cohort, controls selected from the same general population; non telomerase-ALT, GBM negative for telomerase activity and ALT. The number of individuals with each genotype is given in parentheses;

**, p = 0.007, non telomerase-ALT versus telomerase positive, Odds Ratio 0.333, 95% CI 0.156–0.732; p = 0.002, non telomerase-ALT versus telomerase and ALT combined, Odds Ratio, 0.312, 95% CI 0.152–0.643; p = 0.001, non telomerase-ALT versus the non-tumor cohort selected from the same general population, Odds Ratio 0.365, 95% CI 0.2–0.67. All comparisons were tested using the Fisher's exact test p<0.05 was taken as a significant difference.

All individuals with the G500 allele were homozygous for the major allele at two other polymorphic sites in close proximity (C540T and C547G). No *CDKN2A* mutations were identified in G500 heterozygotes as evident by dHPLC and sequence analysis of all *CDKN2A* exons and exon/intron boundaries in amplicons of tumor and blood leukocyte extracted DNA. Nine individuals (3 with telomerase, 1 with an ALT, and 5 with NDTMM tumors) were heterozygous for the G442A polymorphism that substitutes an adenine for a tyrosine amino acid at amino acid residue 148 in p16^INK4a^.

In summary, in GBM the G500 allele and no other *CDKN2A* sequence variant examined was associated with an increased frequency in the NDTMM group. In the telomerase GBM group the G500 allele frequency was not significantly different to the control population selected with the same ethnicity, and age and sex matched to the GBM cohort.

### The G500 allele is associated with poorer survival in individuals with GBMs with no defined telomere maintenance mechanism

Cox proportional hazards regression analysis was performed to test whether the GC and CC C500G genotypes in the NDTMM and telomerase positive groups were independently associated with survival. Variables included in the analysis were age, gender, treatment, and extent of surgery (Table 3). The C500G SNP was significantly associated with survival with the adjusted ratio for survival (p = 0.029, variable 1 = CC, Hazard ratio 0.197, 95% CI 0.046–0.844). Other variables independently associated with altered survival were age (p = 0.037, variable age in years, hazard ratio 1.021, 95% CI 1.001–1.041) and chemotherapy treatment (p = 0.002, variable chemotherapy yes = 1, hazard ratio 0.392, 95% CI 0.220–0.699).

**Table 3:** Cox Proportional Hazards Model for Factors Affecting Survival.

Variable	p	HR (95% CI)
Age (years)	0.037	1.021 (1.001–1.041)
Gender	0.469	1.215 (0.717–2.058)
Tumor resection	0.333	0.735 (0.395–1.371)
Surgery + Radiotherapy	0.393	0.724 (0.346–1.517)
Surgery + Radiotherapy + Chemotherapy	0.002	0.392 (0.220–0.699)
C500G SNP (CC)	0.027	0.197 (0.046–0.844)
NDTMM	0.451	0.558 (0.123–2.541)
p16^INK4A^ loss	0.224	0.419 (0.1–1.755)

HR, Hazard ratio; 95% CI, 95% Hazard ratio confidence limits.

**Figure 1:**
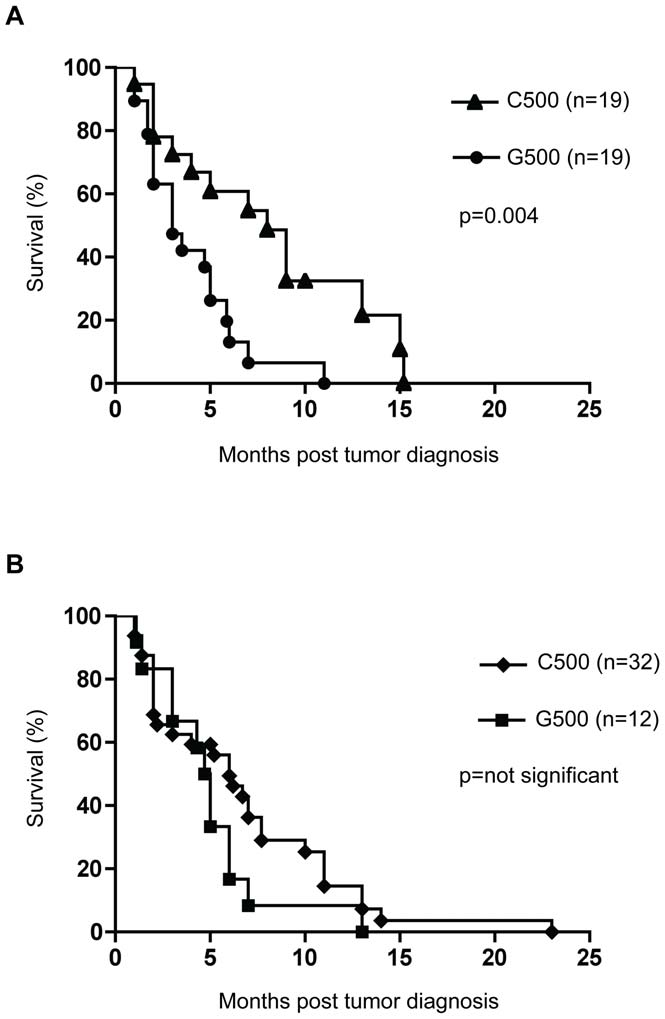
Patient survival with the G500 allele compared to C500 homozygotes for individuals with NDTMM and telomerase positive GBMs. **A.** Kaplan-Meier survival analysis post tumor diagnosis for individuals with GBMs with no defined telomere maintenance mechanism (NDTMM). Survival data was available for all 38 individuals (the G500 homozygotes were excluded from the analysis). **B.** Kaplan-Meier survival analysis post tumor diagnosis for individuals with telomerase positive GBMs. Survival data was available for 44 individuals. Individuals genotyped as heterozygote for the C500G polymorphism (G500) in the 3′ *CDKN2A* UTR were associated with reduced survival compared to C500 homozygotes (C500) using a multi-variant Cox proportional hazards regression analysis (Table 3).

In the NDTMM tumor group survival data was available for all 40 individuals. Individuals heterozygous for the G500 allele had a median survival time of 3 months following the initial tumor diagnosis, compared to 8 months for individuals homozygous for the C500 allele (Figure 1A). The two individuals homozygous for the G500 allele had survival times of 1 and 2 months post tumor diagnosis, respectively. The median age for G500 heterozygotes with a NDTMM GBM was 66 (60–75, 25^th^ to the 75^th^ percentile), which was higher compared to a median age of 58 for C500 homozygotes (41–70, 25^th^ to the 75^th^ percentile), p = 0.04 (two tailed unpaired *t* test with Welch's correction, 95% CI).

In the telomerase positive tumor group survival data was available for 44 of 50 individuals. Individuals heterozygous for the G500 allele had a median survival time of 4.9 months following the initial tumor diagnosis compared to median survival time of 6 months for CC homozygotes (Figure 1B).

### The G500 allele is associated with *p16^INK4a^* gene loss in GBMs with no defined telomere maintenance mechanism

To test if the reduced survival associated with the G500 allele in the NDTMM GBM cohort was attributed to a greater loss of homozygosity or heterozygosity for *p16^INK4a^* or *p14^ARF^*, multiplex PCR was used to estimate the gene dosage of exon 1α (*p16^INK2A^)* and exon 1β (*p14^ARF^)* relative to an internal β-globin gene fragment [20]. In the NDTMM group, 15 out of 19 G500 heterozygotes had exon 1α and exon 1β loss of homozygosity or loss of heterozygosity, and 4 retained exon 1α and exon 1β homozygosity. In C500 homozygotes, 7 individuals had exon 1α and exon 1β loss of homozygosity or loss of heterozygosity, and 12 retained exon 1α and exon 1β homozygosity. The increased frequency of exon 1α and of exon 1β loss in G500 carriers compared to C500 homozygotes was significant (p = 0.02).

**Figure 2:**
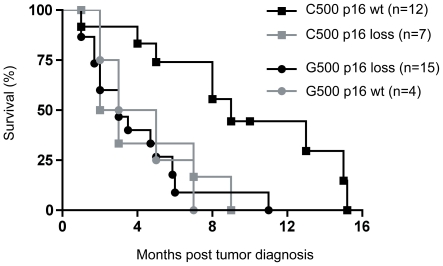
The improved prognosis for C500 homozygotes is associated with retention of *p16^INK4a^ and p14^ARF^*. Kaplan-Meier survival analysis from Figure 1A with individuals with GBMs with no defined telomere maintenance mechanism further divided on exon 1α *(p16^INK4A^)* gene dosage. Individuals homozygous for C500 were divided into those that had loss of homozygosity or loss of heterozygosity (C500 p16 loss) and those that retained exon 1α homozygosity (C500 p16 wt) by multiplex PCR of tumor DNA. The same division was made for G500 heterozygotes (G500 p16 loss and G500 p16 wt). The C500 p16 loss group had a significantly poorer survival to the C500 p16 wt group (p = 0.016, log-rank test with 95% CI). No difference in survival occurred for the G500 p16 loss and G500 p16 wt comparison. All associations for *p16^INK4a^* pertained to *p14^ARF^* due to identical exon 1α and exon 1β gene dosage.

In the NDTMM group, individuals heterozygous for G500 that had loss of exon 1α and 1β gene dosage had a median survival of 4 months, which was not significantly different to G500 heterozyotes that retained exon 1α and 1β homozygosity (median survival 3 months, Figure 2). However, the few individuals that retained gene dosage limit this comparison. Individuals homozygous for the C500 allele that had loss of exon 1α and 1β gene dosage had a median survival of 2.5 months, which was significantly reduced compared to those that retained exon 1α and 1β homozygosity (median survival 9 months, p = 0.016 hazard ratio 3.3, Figure 2).

Cox proportional hazards regression analysis was performed to test whether loss of p16^INK4a^ was an independent variable associated with survival. Loss of p16^INK4a^ was not associated (p = 0.419) with altered survival when adjusted for other variables (Table 3).

In summary, in NDTMM GBMs with the G500 allele had increased loss of exon 1α and 1β. Improved survival was found with NDTMM GBMs homozygote for C500 and no loss of exon 1α and 1β; however, loss of p16^INK4a^ was not and independently associated with survival when multiple variables were analyzed.

To verify that the NDTMM GBMs had lost the *p16^INK4a^* gene, tumor sections were analyzed using a fluorescent in situ hybridization [21] and immunohistochemistry assays. For the FISH analysis, sections were incubated with a probe to the *p16^INK4a^* locus and a control probe to chromosome 9 labeled with a different fluorophore (P16 Deletion Probe, Cytocell Ltd, Cambridge UK). The 20 tumor sections selected included ten from G500 heterozygotes typed with loss of *p16^INK4a^* gene dosage using the multiplex PCR analysis and ten tumors from C500 homozygotes typed with no loss in *p16^INK4a^* gene dosage by multiplex PCR analysis. All tumors from C500 homozygotes had 2 red and 2 green signals in cellular nuclei, a result consistent with no loss in *p16^INK4a^* gene dosage (data not shown). Seven tumors from G500 heterozygotes had 2 green signals and no red signals consistent with a loss of both *p16^INK4a^* alleles (data not shown). Three tumors from G500 heterozygotes had 2 green signals and 1 red signal consistent with a loss of one *p16^INK4a^* allele. The results from the FISH analysis confirmed those obtained using PCR.

**Figure 3:**
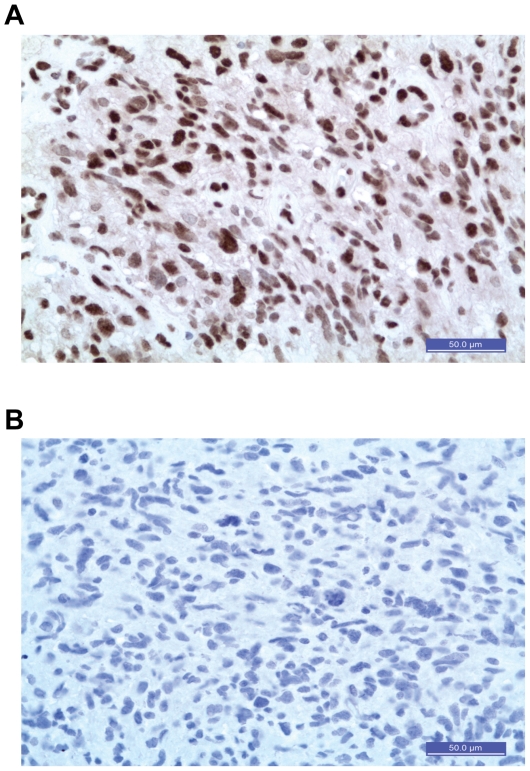
Tumors with the non defined telomere maintenance mechanism with both p16*^INK4a^* alleles produce p16^INK4a^. All tumors used to confirm the presence or losses of *p16^INK4A^* gene dosage by fluorescent *in situ* hybridization [21] were analyzed by immunohistochemistry for the p16^INK4A^ protein. **A.** Photomicrographs to illustrate tumor cells positive for p16^INK4A^ in a GBM typed with both *p16^INK4A^* alleles by FISH. **B.** Photomicrographs to illustrate tumor cells negative for p16^INK4A^ in a GBM typed with loss of both p*16^INK4A^* alleles by FISH. DAB positive cells were detected by light microscopy at 400× magnification, scale bars are included.

For the immunohistochemistry analysis, sections were incubated with an antibody to p16^INK4a^. The 20 tumor sections selected were those used for the FISH analysis described above. All tumors from C500 homozygotes had p16^INK4a^ positive cells (Figure 3A), a result consistent with *p16^INK4a^* expression. Seven tumors from G500 heterozygotes had no p16^INK4a^ positive cells, consistent with loss of both *p16^INK4a^* alleles (Figure 3B).

In the telomerase positive and ALT positive groups no significant difference occurred for exon 1α and 1β gene dosage between G500 heterozygotes and C500 homozygotes using the multiplex PCR. In telomerase positive tumors, 9 out of 12 tumors from G500 heterozygotes had loss of homozygosity or loss of heterozygosity for exon 1α and 1β, 1 had lost exon 1α but retained exon 1β gene dosage, and 2 retained both exon 1α and exon 1β gene dosage. In C500 homozygotes with telomerase positive tumors, 20 out of 38 tumors had loss of homozygosity or loss of heterozygosity for exon 1α and 1β, 2 had lost exon 1α but retained exon 1β gene dosage, 2 had lost exon 1β but retained exon 1α gene dosage, and 9 retained both exon 1α and exon 1β gene dosage. In ALT positive GBMs, 3 out of 3 tumors from G500 heterozygotes had loss of heterozygosity for exon 1α and 1β. In C500 homozygotes with ALT positive tumors, 9 out of 14 tumors had loss of homozygosity or loss of heterozygosity for exon 1α and 1β, and 5 retained both exon 1α and exon 1β gene dosage.

### Isocitrate dehydrogenase 1 mutations are associated with ALT positive GBMs

Tumor DNA from all GBMs was used as the template in PCR reactions that amplified all exons of *IDH1* and exon 4 of *IDH2*. Sequence analysis was used to identify missense mutations. Twelve GBMs were heterozygote for missense mutations in *IDH1*. Eleven individuals carried the R132H mutation; the most frequent IDH1 missense mutation documented for GBM [22], [23]. One individual carried the R132L mutation also reported for GBM [24]. Ten tumors with IDH1 missense mutations were ALT positive, thus IDH1 mutations were present in 59% (10 of 17) of the ALT cohort and 11% of total GBMs. Mutations in IDH1 were present in one telomerase positive (2%) and one NDTMM (2.5%) tumors. The frequency of IDH1 mutations in ALT was significant when compared to both telomerase and NDTMM tumors (Pearson's chi square test p<0.001 for both the comparison of ALT with telomerase, and ALT with NDMM tumors). All individuals with R132H mutations were under 45 years of age, with one exception the individual with the telomerase positive tumor. In the ALT positive tumor cohort, the younger age of individuals with IDH1 mutations was significant compared with those without IDH1 mutations (median age 36 versus 52, 25^th^ to the 75^th^ percentile 26–40.5 versus 48–61, p<0.001). For IDH2 mutations, one mutation was found. The D177A, amino acid residue substitution occurred in a NDTMM GBM.

In summary, the majority of ALT positive tumors contained IDH1 mutations. In NDTMM and telomerase positive GBMs, IDH1 mutations were rare.

## Discussion

Currently there is no prognostic marker for GBM tumors without a defined telomere maintenance mechanism. The G500 *CDKN2A* allele may be an important biomarker for NDTMM GBMs identifying individuals with a poorer prognosis. The C500G genotype was associated with survival independently of age and treatment, which are other factors associated with GBM patient survival [6], [21], [25], [26].

It is unknown why a considerable portion of GBMs have no currently defined telomere maintenance mechanism. Most tumor types documented without telomerase activity or ALT are low-grade tumors such as grade 1 astrocytomas and papillary thyroid carcinomas [8], [27]. With few exceptions telomerase activity is measured using the TRAP assay, which is sensitive for detecting telomerase activity in 0.01% of telomerase positive cells in a mixed population [5], [7], [28]. Inhibitors of the TRAP assay in tumor cell lysates may produce false negative results [29]. In this study, measuring telomerase activity using at least four different dilutions of each lysate reduced this limitation of the TRAP assay inhibitors. All tumors classified as ALT negative had mean TRF lengths below 13 kbp and did not show a heterogeneous TRF profile. Non-defined telomere maintenance mechanism tumors had fewer *TP53* mutations compared to ALT positive tumors (unpublished observations). Moreover, the different G500 allele frequency, the absence of *IDH1* mutations, and the different effect of mutant *TP53* on survival (also replicated in this GBM cohort, data not shown) on survival mitigates against these “None” GBMs being miss-classified as telomerase or ALT tumors [7]. Our data suggest that the NDTMM tumors are a distinctive subgroup of GBMs. The G500 allele was associated with an increased age in heterozygotes compared to C500 homozygotes in the NDTMM group, therefore the age and composition of GBM cohorts may affect the NDTMM tumors present.

The G500 allele as a risk factor for cancer has been most extensively characterized for melanoma [12], [13], [30]. The G500 allele had an increased frequency in patients from families with a high risk of melanoma [12]. How the G500 allele affects cancer risk is unknown, but may lead to loss of *p16^INK4a^* and *p14^ARF^* expression [16] [15]. Deletion of *CDKN2A* is associated with poor tumor outcome in patients and mice [31], [32], [33]. The mechanism by which the G500 allele leads to a loss of exon 1α and exon 1β in NDTMM GBMs is unclear and not immediately intuitive, and requires further investigation. One could predict that a C500G base substitution in a 3′ UTR could alter the stability of the *p16^INK4a^* and *p14^ARF^* transcripts, which may give a greater selection pressure for inactivation by a later DNA deletion [34]. MiR-601 binding to *CDKN2A* was predicted to be affected by the C500G polymorphism [18].

The G500 allele was not associated with another sequence variant in the *CDKN2A* coding region or the 3′ UTR of *CDKN2A* region analyzed. The C500G alleles may affect expression of other genes in close proximity to *CDKN2A*. Both *CDKN2B* and the non-coding RNA designated *ANRIL* are included with *CDKN2A* in the susceptibility locus associated with glioma and other diseases [35], [36], [37], [38]. According to Cunnington *et al*. (2010), the G500 allele reduced *CDKN2B* and no effect on *ANRIL* expression [19].

The causative variant may be in linkage disequilibrium with the G500 allele and in a region on chromosome 9 not analyzed in this study.

Homozygotes for the C500 allele with loss of exon 1α and exon 1β gene dosage had a poorer survival compared to those that had retained exon 1α and exon 1β, consistent with an association between loss of *p16^INK4a^* and *p14^ARF^* function and poorer survival. Interestingly, this was not the case for G500 heterozygotes, as those that had loss of exon 1α and exon 1β gene dosage had no difference in survival compared to those that retained exon 1α and exon 1β. One explanation for this discrepancy is that the G500 allele may lead to loss of *p16^INK4a^* and *p14^ARF^* transcripts initially, followed by loss of exon 1α and exon 1β DNA at a latter stage of tumor development. The poorer survival associated with the G500 allele may be attributed in part to an increased age at initial tumor diagnosis for G500 heterozygotes [39]. Tumor surveillance and genome protection may be compromised by age leading to a more rapidly fatal tumor. Although the C500 allele is associated with improved prognosis in NDTMM GBMs, its association with a younger age is indicative that it could be considered a risk allele for this GBM type. The C500 allele was the risk factor for Alzheimer's disease [40]. A greater understanding of the function of each allele is required to ascertain how the base change leads to increased disease susceptibility.

The NDTMM GBMs were not associated with *IDH1* mutations, a marker for improved prognosis in ALT positive tumors [6]. Consistent with another report, IDH1 mutations in the current study were associated with ALT positive GBMs. The percentage of ALT positive GBMs (59%) in the current study was higher than that previous reported (18.8%) [6]. This difference could be attributed to the different techniques used to identify IDH1 mutations (sequencing versus immmunohistochemistry used in the previous report) or a difference in the age of the ALT positive GBM cohort (median age 36 versus 50 in the previous report). GBMs with IDH1 mutations are associated with reduced age for affected individuals, and in our ALT positive tumor cohort all individuals with IDH1 mutations were aged under 45 [23], [41]. Mutations in IDH1 are thought to occur early in tumorigenesis, with recent evidence suggesting IDH1 mutations may have tumor promoting functions [42], [43]. In glioma IDH1 mutations are associated with a CpG methylator phenotype [44]. In acute myeloid leukemia IDH1 mutations have been proposed to increase global hypermethylation by blocking the function of the tet oncogene family member 2 function [45]. The molecular mechanism by which *IDH1* mutations and ALT are associated in Glioma remains to be understood.

New treatments targeting telomerase positive tumors are being developed [46]. If successful, an analysis of tumor telomere maintenance mechanisms may be important to identify individuals that would benefit from telomerase inhibitors. Other treatment approaches may be required for those with ALT positive and tumors with no defined telomere maintenance mechanism.

### Conclusion

The *CDKN2A* G500 allele in the 3′ UTR was more frequent in patients with tumors that have no defined telomere maintenance mechanism and was a prognostic marker for poorer survival. The increased incidence of the G500 allele and the absence of IDH1 mutations provide justification for the NDTMM GBMs being a molecularly distinct subgroup of tumor. Further investigation is required to understand the function of the G500 allele in altered cancer risk. In younger GBM patients with ALT positive tumors, IDH1 mutations predominate. Therefore the molecular signature of telomere maintenance mechanisms may provide additional prognostic information for GBM patients in addition to that for age and treatment received.

## Materials and Methods

### Ethics Statement

The study had ethical approval from the Multi-region Ethics and the Upper South Regional Ethics committees in New Zealand, and written individual informed consent was obtained.

### Tumor Collection

The inclusion criterion for the study was a diagnosis of a GBM tumor. One hundred and seven tumors with both paraffin-embedded and frozen tissue available were analyzed. All tumors were collected in New Zealand, and all were debulking samples. Consultant pathologists performed the histopathological tumor diagnoses. All individuals were of New Zealand European ethnicity.

### Telomere Maintenance Mechanism Analyzes

Telomerase activity was measured in tumor lysates using the Telomeric Repeat Amplification Protocol (TRAP) method. The TeloTAGGG Telomerase PCR ELISA Plus kit (Roche Applied Science, Mannheim, Germany) was used according to the manufacturer's instructions. For identification of ALT positive tumors, measurement of telomere length was made by the terminal restriction fragment (TRF) assay using tumor lysates. The TeloTAGGG Telomere Length Assay Kit (Roche Applied Science, Mannheim, Germany) was used according to the manufacturer's instructions. The criteria for ALT by TRF length was that previously described [8]: mean TRF length >16 kbp, with a wide range in TRF lengths (typically <3 to >50 kbp). Determination of ALT was also made by measurement APBs. Co-localized bodies of PML and telomere DNA, in cellular nuclei, were performed on sectioned paraffin embedded tumors and determined according to the APB assay criteria described previously [8]. Cellular nuclei were stained using DAPI. Cells were imaged using confocal microscopy (Zeiss LSM510; Carl Zeiss, NY USA), and images analyzed by Zeiss LSM Image Examiner software version 3.2.0.115 (Carl Zeiss, NY USA).

### Genetic analyses of *CDKN2A*

#### C500G polymorphism genotyping

To genotype the C500G (rs11515) polymorphism [47], a 435 bp region inclusive of C500G, and two other single nucleotide polymorphisms in close proximity (C540T rs3088440 and C547G rs79294022) were amplified from DNA extracted from blood leukocytes followed by sequencing. The PCR reaction used a sense primer in intron 2 (5′-GTGCCACACATCTTTGACCTCAG-3′) and an anti-sense prime in the 3′ UTR (5′-TGCTTGTCATGAAGTCGACAGCT-3′). The C500G alleles were genotyped in 150 blood leukocyte extracted DNA samples from individuals randomly selected from the general New Zealand population of European ancestry.

#### CDKN2A coding region amplification

All exons of *CDKN2A* were amplified by PCR using blood leukocyte and tumor extracted DNA as the template. The following sets of primers were used to amplify exon 1α (sense 5′-ACCGGAGGAAGAAAGAGGAG-3′, antisense 5′-AGTCGCCCGCCATCCCCT-3′) and 1β (sense 5′-CGCTCAGGGAAGGCGGGTGCGCG-3′, antisense 5′-CCTAGCCTGGGCTAGAGACG-3′) [48]. Primers sequences for amplification of exon 2 and 3 were those previously described [49]. All PCR products were screened for sequence variants by denaturing HPLC (d-HPLC) using the WAVE system (Transgenomic, NE, USA), and sequenced (ABI3730 DNA Analyzer, AB Applied Biosystems, CA, USA) if aberrant dHPLC profiles indicative of sequence variation presented. If sequence variants were not predicted, PCR products were mixed with *CDKN2A* wild-type DNA (1∶1) and re-analyzed by dHPLC to allow non-heterozygous sequence variants to be detected.

#### CDKN2A gene dosage using multiplex PCR

Multiplex PCR as previously described for astrocytomas, was used to assay the gene dosage of *CDKN2A* exon 1α (*p16^INK4A^*) and exon 1β (*p14^ARF^*) using tumor extracted DNA [20]. The multiplex PCR designed to amplify exon 1α and that for exon 1β amplified part of the β-Globin gene as an internal control. PCR product intensities were analyzed by gel electrophoresis and used Bio-Rad Quantity One (Bio-Rad Laboratories, CA, USA) and Syngene GeneTools image software (Syngene, Cambridge, UK).

#### p16^INK4a^ gene dosage using fluorescent in situ hybridization

*In situ* hybridization was used to confirm the p16^INK4a^ gene dosage results obtained from the multiplex PCR method, and measured p16^INK4a^ gene dosage in 20 NDTMM GBM paraffin embedded tumor sections using the P16 Detection Probe (Cytocell Aquarius, Cambridge, UK) and the control chromosome 9 probe deletion probe (D9Z3 Cytocell Aquarius). The *in situ* hybridization protocol was based on that used in the laboratory for FISH analysis of telomeric DNA [50]. Cellular nuclei were stained using DAPI. Cells were imaged using microscopy (Zeiss LSM510; Carl Zeiss, NY USA), and analyzed by Zeiss LSM Image Examiner software version 3.2.0.115 (Carl Zeiss) to identify tumor cell nuclei with 1, 2, or no red dots, and two green dots.

### Genetic analyses of *IDH1 and IDH2*

To identify tumors with IDH1 mutations all exons of the IDH1 gene were amplified from tumor extracted DNA and sequenced. With the exception of the PCR reaction designed to amplify exon 4, which used the following set of primers, sense 5′-ATGCCATCACTGCAGTTGTAGG-3′ and antisense 5′-CCTTGCTTAATGGGTGTAGAT-3′, all primers used were those described previously [9]. The analysis of IDH2 mutations was limited to an analysis of exon 4, which was amplified from tumor DNA using previously described primer sequences and PCR products sequenced [9].

### p16^INK4a^ immunohistochemistry

The p16^INK4a^ epitope was detected using an anti-mouse p16^INK4a^ monoclonal antibody (ab454210, Abcam, Cambridge. MA), and detection of the enzyme linked conjugated system (ENVision Dual Link, Dako, Glostrup, Denmark) used DAB. Detection of DAB positive cells used a Leica DM2000 light microscope at 400× magnification and Leica Version 3.5.0 Application Suite software (Leica Microsystems, Wetzlar, Germany).

### Survival and Statistical Analyses

The primary end point was overall survival. Cox proportional hazards regression analysis was performed to assess the GC and CC G500C genotypes as a prognostic marker for survival and was adjusted for other variables age (years), gender, tumor resection (biopsy only, debulking), and treatment (radiotherapy yes or no, chemotherapy yes or no), loss of p16^INK4a^. The analysis was made using the PHREG Procedure SAS System (Local, WIN_PRO, SAS Institute, Cary, NC).The frequency of the G500 allele was compared between GBMs with different telomere maintenance mechanisms using the Fisher's exact test and GraphPad Prism software (GraphPad Software, CA, USA). In the NDTMM and telomerase GBM groups the age at brain tumor diagnosis was compared between G500 heterozygotes and C500 homozygotes, and used the unpaired Student's *t* test with 95% CI and Welch's correction to compensate for unequal variance. In the NDTMM and telomerase GBM groups, the loss of homozygosity or the loss of heterozygosity of exon 1α or exon 1β was compared to tumors that retained exon 1α or exon 1β homozygosity using the Fisher's exact test and GraphPad Prism software (GraphPad). The frequency of missense mutations in *IDH1* was compared between GBMs with different telomere maintenance mechanisms using the Pearson's chi square test and SHEsis software [51]. A p<0.05 was taken as a significant difference. The association between age, telomere maintenance mechanism, and IDH1 mutation status was analysed using the analysis of variance test and used GraphPad Prism software (dependent variable being age).
